# Air Core ARROW Waveguides Fabricated in a Membrane-Covered Trench

**DOI:** 10.3390/photonics11060502

**Published:** 2024-05-25

**Authors:** Seth Walker, Holger Schmidt, Aaron R. Hawkins

**Affiliations:** 1 Electrical and Computer Engineering Department, Brigham Young University, 450 Engineering Building, Provo, UT 84602, USA; 2 School of Engineering, University of California, Santa Cruz, CA 95064, USA

**Keywords:** waveguides, microfabrication, integrated optics, photonics

## Abstract

We report the design, fabrication, and characterization of hollow-core anti-resonant reflecting optical waveguides (ARROWs) fabricated in a membrane-covered trench. These structures are built on silicon wafers using standard microfabrication techniques, including plasma etching, to form trenches. Four waveguide designs are demonstrated, which have different numbers of thin-film reflecting layers. We demonstrate that optical loss decreases with additional reflecting layers, with measured loss coefficients as low as 1 cm^−1^.

## 1. Introduction

Many fields of study use light interactions with gases and liquids [1]. Frequently, confined high intensity light is needed for these interactions [2]. Using a hollow waveguide, while confining light to a mode, allows for a larger range of interaction distances to be used with higher optical intensities [3,4]. Standard waveguide performance specifications include optical loss coefficient and optical mode size. These metrics help determine if a hollow waveguide is useful for a given application. Hollow waveguides have found applications ranging from gas and chemical sensing [5], quantum and nonlinear optics [6], MEMS sensors [7], hollow core fibers [8], and free-space optical communications systems [9].

Low-loss light propagation in hollow waveguides presents many challenges. Total internal reflection (TIR), the process in which light is confined to a high-refractive index region surrounded by lower index cladding, is typically used to achieve propagation in classical waveguides [10,11]. However, hollow-core waveguides have cores with the lowest possible refractive index of 1. No cladding materials exist for the construction of TIR-based hollow core waveguides, so other designs must be employed. One type of hollow-core waveguide that has been demonstrated effectively is the anti-resonant reflecting optical waveguide, or ARROW. Typically, one or more anti-resonant layers are used to surround the guiding core [12]. With these layers, light is reflected toward the core at thin-film interfaces which have alternating indices of refraction. Often, designs built on silicon wafers depend on the wafer bonding of two wafers with ARROW layers already deposited on the surfaces or building the waveguides on a raised pedestal to elevate the waveguides from the surface of the wafer. An ARROW mode still exhibits loss but can be designed to achieve low-loss propagation over long distances. Typical designs use single-mode operation. These designs have been used in many applications, ranging from optical trapping [13], bio sensing [14], and on-chip rubidium spectroscopy [15].

In this paper, we propose and demonstrate an ARROW structure that combines an etched trench with a thin top membrane. ARROW layers are used as a membrane and a rib structure is built into this top membrane to enable horizontal mode confinement. This confinement will reduce interaction with the sidewalls, reducing overall optical loss. A thin top membrane is useful in various MEMS applications such as pressure and vibrating sensors, which also utilize light interactions. The design utilizes standard silicon device processing. We describe the design objectives, fabrication process, and optical characterization results.

## 2. Waveguide Design

For our design, we explored using a trench instead of a pedestal because we wanted our device to be simple to build and easy to integrate into various other photonic designs. A trench can simplify the interaction between our waveguide and other optical elements. It also avoids raised topography, which typically makes subsequent lithography steps and surface fabrication difficult. We also wanted to avoid wafer bonding so that our waveguide could be easily accessible on a wafer’s surface.

Our first design consideration involved the thin films meant to act as ARROW layer pairs. Besides being compatible with silicon microfabrication processes, our layers must have large differences in refractive indices and be optically transparent to the light meant to propagate down the waveguide. Previous work carried out by our group demonstrated ARROWs utilizing PECVD-grown silicon dioxide (refractive index ≅1.5) and silicon nitride (refractive index ≅2.0) [16]. Using this as a foundation, these materials were again chosen to prove our new design. The correct thickness, di, for each thin-film layer to create a Fabry–Perot reflector can be determined by Equation (1) [17].


(1) $$d_{i}=(2N-1)\frac{\lambda }{4n_{i}\sqrt{1\;-\;\frac{{n_{c}}^{2}}{{n_{i}}^{2}}\;+\;\frac{\lambda^{2}}{4{n_{i}}^{2}{d_{c}}^{2}}}}$$

**Figure 1.**
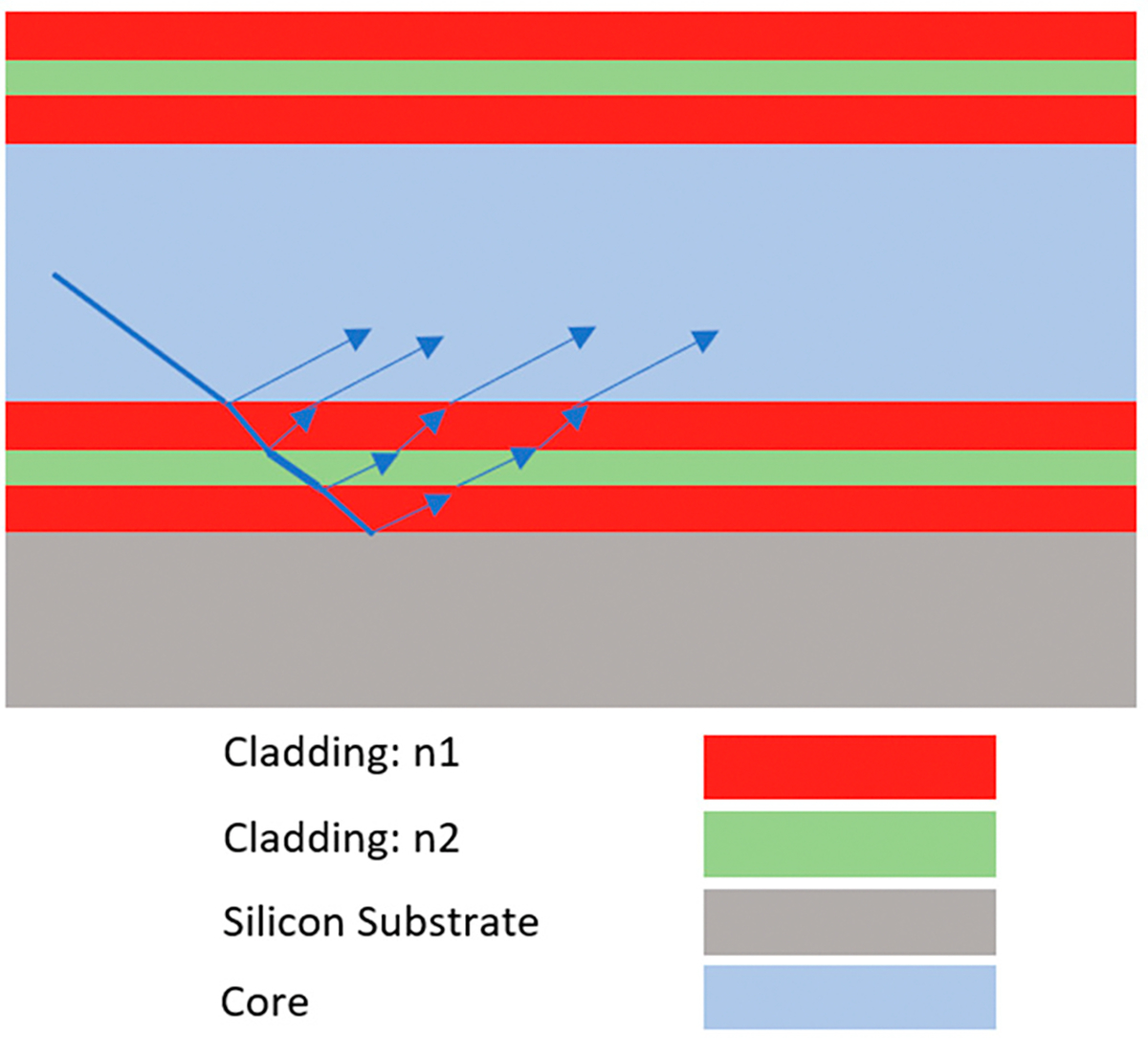
Illustration of a longitudinal cross-section for a hollow-core waveguide. ARROW layers are depicted, as well as how they are designed to guide the light back to the core region.

In this equation, ni and nc are the index of refraction for the thin-film ARROW layer and core, respectively, λ is the wavelength of light, N is an integer representing the antiresonance order, and dc is the thickness of the core. An example of an ARROW device geometry is shown in Figure 1.

Based on trench geometries achievable with contact photolithography and the deep-reactive ion etching of silicon, we designed for trench widths 20 and 30 μm wide and 15 μm tall. By targeting 650 nm laser light and setting N = 0 in Equation (1), we calculated the necessary thickness for the silicon nitride and silicon dioxide layers to be 91 nm and 145 nm, respectively [16]. These layer thicknesses can be stacked together, so these values were used in our multiple-layer designs. We also built a single silicon dioxide layer design, which would act like a reflector. We experimentally iterated through three different thicknesses to find the best option. At 200 nm, our devices did not survive the final etching process, and at 400 nm, there was essentially zero throughput. Finally, at 300 nm, we measured low-loss propagation and determined this would be the ideal thickness.

**Figure 2.**
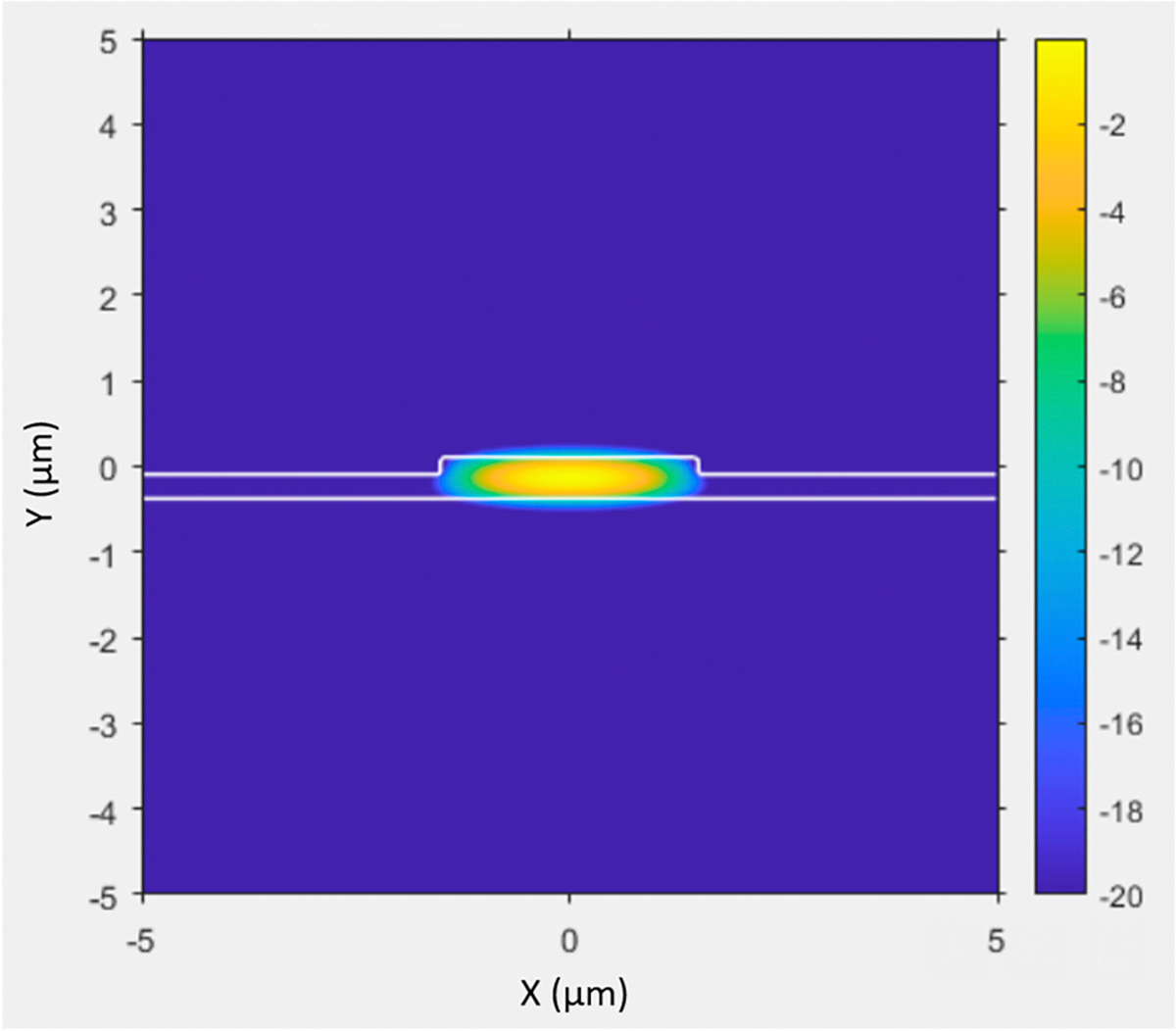
Simulation of a rib and how it would act as a horizontal confinement for the light. The most intense region starts at yellow and decreases to blue.

The second design consideration was the horizontal confinement of light away from the trench sidewalls. Fabrication can create various imperfections, causing scattering and high optical loss. An example of this are the scallop features which generally form on the side walls during deep plasma etching in silicon. These features result in high optical scattering loss when encountered by light. The trench being composed of silicon, which absorbs at 650 nm and has a very high refractive index, would also contribute to high optical loss. To reduce light interaction with these walls, we added a 5 μm wide, 300 nm tall rib of SiO_2_ to span the entire channel length with the ARROW layers grown over the top of it. Figure 2 is a simulation of this rib structure. This rib was meant to horizontally confine light through TIR. The addition of the rib creates a hybrid waveguide structure that combines elements of TIR and ARROW light guiding.

## 3. Fabrication

After determining optimal design parameters to minimize optical loss, we developed several new fabrication processes. We wanted to borrow from previous fabrication processes used for ARROWs on self-aligned-pedestals and planar substrates that used PECVD and sacrificial etching [17–19], but our new hybrid structure had a new set of challenges. First, we needed a sacrificial core that would fill up a trench structure and allow for the deposition of a robust membrane. We determined that a capillary-filling method utilizing SU8, a photo-definable polymer [20], was the best way to create a sacrificial core, but this left a second challenge of removing large SU8 droplets used for trench filling. A third challenge was defining a rib on top of our sacrificial core that would be small enough to be positioned as far as possible from the trench walls.

**Figure 3.**
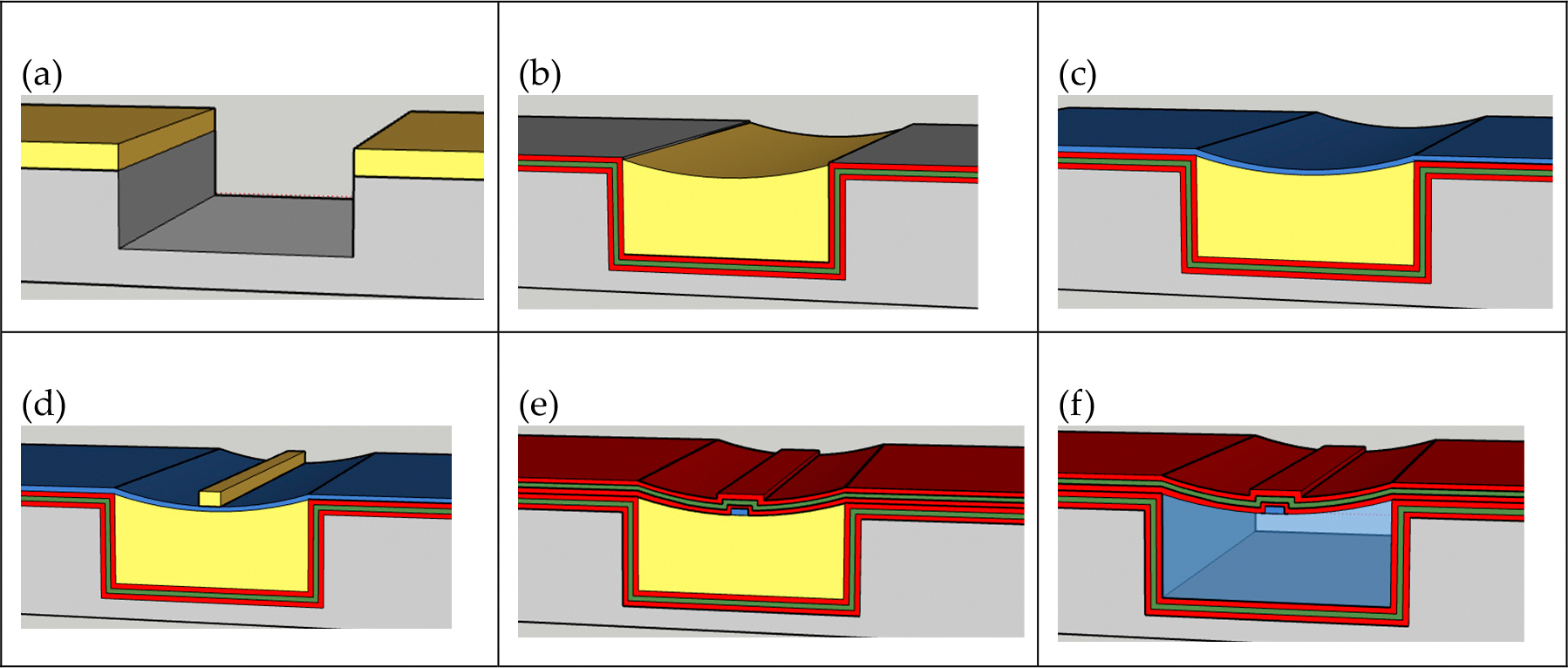
Outline of trenched ARROW design fabrication process. (**a**) Silicon trench etch. (**b**) ARROW layer deposition and SU8 droplets. Red and green are used to highlight the difference in index of refraction of each layer. (**c**) Thin oxide for TIR rib. (**d**) Pattern and etch rib. (**e**) ARROW layer deposition. (**f**) SU8 removal.

Our final fabrication process utilized the following major steps. Step (1): A 2 μm thick photoresist (AZ 2020) layer was deposited onto the silicon substrate and patterned into 10 mm long channels with widths of 20 and 30 μm. Reservoirs were then patterned in between each 10 mm long device that would allow for the sacrificial core to fill subsequent trenches through capillary action. Step (2): The silicon was then etched using an Inductively Coupled Plasma (ICP) Reactive Ion Etcher (RIE) employing the following gases: SF6, O2, and C4F8. Our targeted etch depths were 15 μm [see Figure 3a].

At this point, we created four designs based on different ARROW pairs deposited on the wafer using PECVD. We used one machine specifically for the oxide layers and one machine for the nitride layers. After each layer was deposited, the machine was cleaned to ensure the next deposition would be accurate. Step (3): For design 1, we simply deposited 300 nm of oxide. For design 2, we deposited an additional ARROW layer pair, that is, 140–150 nm of oxide, 91 nm of nitride, and another 140–150 nm of oxide. For design 3, we deposited 2 additional ARROW layer pairs, and for design 4, we deposited 3 additional ARROW layer pairs. Step (4): A droplet of the sacrificial core, SU8–2000.5, was then placed into the reservoirs and allowed to flow through the channels through capillary action. Because the deposited PECVD silicon dioxide has a high surface tension, we were able to create a sacrificial core 10 mm long [see Figure 3b]. Step (5): In total, 300 nm of silicon dioxide was then deposited over the channels [see Figure 3c]. Step (6): Every region on the wafer was then covered in photoresist (AZ 4620) except the reservoir regions. Using HF, we were able to remove the silicon dioxide and then selectively etch the reservoir away using a solution of H_2_O_2_ and H_2_SO_4_ at 90 °C. This helped eliminate any effects their presence would have on future lithography processes.

Creating the TIR rib demanded high-precision photolithography alignment. We needed the rib to fit within the width of the etched trench throughout its length. Step (7): Etching into the top layer of oxide over the sacrificial core with hydrofluoric acid, we were able to create a 4–6 μm wide rib [see Figure 3d]. Step (8): For design 1, a final layer of 300 nm of oxide was then deposited over all the trenches. For designs 2, 3, and 4, we deposited 1, 2, and 3 more additional ARROW layer pairs, respectively [see Figure 3e]. Step (9): Devices were then cleaved and placed into a solution of H_2_O_2_ and H_2_SO_4_ at 90 °C to remove the sacrificial cores [see Figure 3f]. After the fabrication process was completed, we used a diamond scribe to cleave our devices to form end facets. To make cleaving as simple as possible, we aligned our trenches orthogonal to the <011> plane.

**Figure 4.**
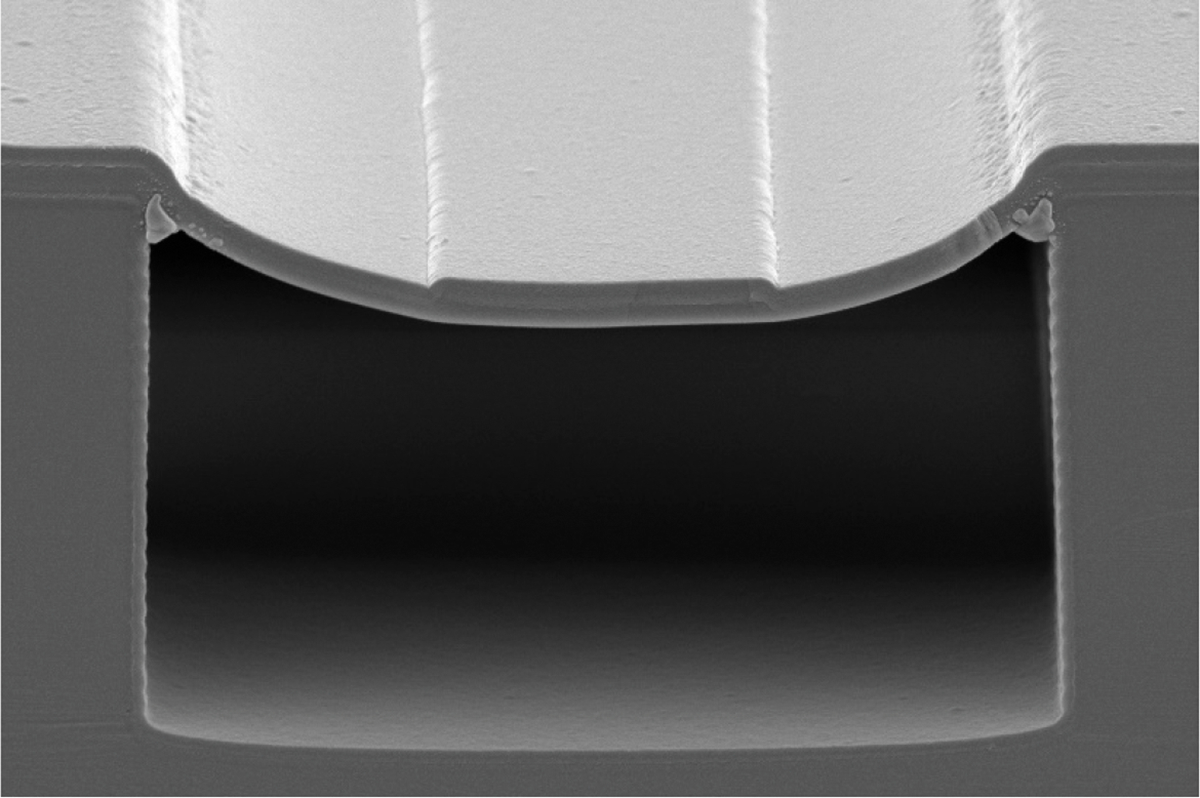
SEM image of a 20 μm wide device with a single ARROW layer pair.

In Figure 4, an SEM image shows the final result of our design. This image highlights the single ARROW layer pair deposited, the rib, and the contrast between the scalloped sidewalls of the trench and the extremely smooth bottom surface. This is common in deep RIE silicon etching. The growth conditions and conformality of the PECVD films in such a deep trench dictate that the sidewalls will have a very different film thickness than that deposited at the bottom of the trench or on any other flat surface of the wafer. Because the sidewalls are rough and have thicknesses that do not match the condition in Equation (1), they are ineffective at contributing to waveguiding. This makes the TIR rib and the horizontal confinement it provides even more critical to achieve low-loss waveguiding.

## 4. Optical Characterization

Before any optical characterization, completed waveguides were inspected through an optical microscope to ensure their facets were straight and defect-free. Waveguides were then illuminated by butt coupling them to a single-mode optical fiber pigtailed to a 650 nm laser. Two tests were performed for a waveguide of a given length, namely (1) measuring total optical transmission and (2) imaging the optical mode profile after light propagated through the waveguide. Transmission values for waveguides cleaved to different lengths were used to determine an optical loss coefficient (cutback method).

Our optical test bench consisted of a three-axis stage that allowed our devices to be moved easily throughout the alignment and testing process. We used an overhead camera to aid in alignment between the waveguide and fiber. After light was transmitted through the waveguides, it went through an objective lens for collection. A mirror was used to either direct the light to a Thorlabs camera so that a mode image could be captured, or to a photodiode so optical intensity could be measured. After collecting a mode image, we used a MATLAB script to process the image. Using the intensity of each pixel, we were able to accurately obtain the height and width of the mode profile. We then created a contour plot of our image. This helped show the intensity profile in our mode and displayed how the light was being guided in the hollow and rib regions of our device.

**Figure 5.**
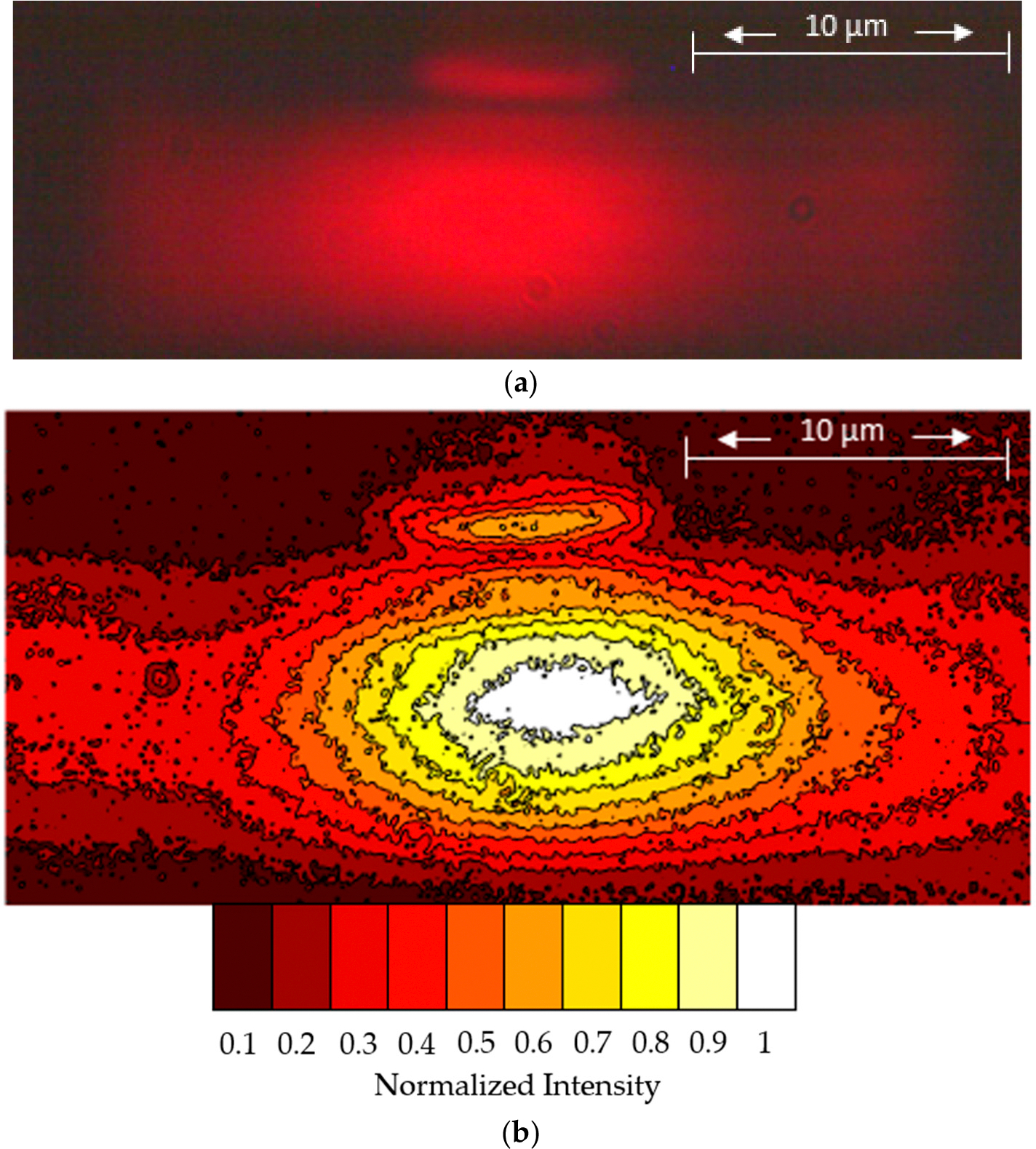
Mode Images. (**a**) A 30 μm device with rib and air core guiding. (**b**) Contour plot of the same image. The center is white which shows the highest intensity of light. Ten bins were used with equally divided intensity in each one. Each succeeding color bin thereafter has less light in it than the first.

In Figure 5, we show the mode profile for light exiting our waveguide. To characterize the size of our modes, we used the full-width-half-max of the intensity, which has a very close match to Gaussian profiles. There is some light contained in the rib at the top of the membrane, but our reported mode measurements are only for the light inside of the dimensions of the hollow trench. In the vertical direction, sizes ranged from 9 to 10.5 μm tall across all four designs and for both 20 and 30 μm wide trenches. As ARROW layer pairs were added, we did see the mode size shrink slightly in this direction. The thin oxide layer and single ARROW layer pair both measured vertically between 9.5–10.5 μm, while the two and three ARROW layer pairs measured around 9 μm tall.

In the horizontal direction, we measured the mode to range from 14 to 16 μm wide across all four designs and for both 20 and 30 μm wide trenches. This shows the effectiveness of the rib and its ability to confine light horizontally, even as the trench size is widened. The modes for the thin oxide layer and single ARROW layer pair designs both measured horizontally around 16 μm. With the two and three ARROW layer pairs, the mode size was around 14 to 15 μm wide. These size changes were in part due to adding more ARROW layer pairs and slightly changing the hollow cross-section size.

We also built two other structures to test our theory about the necessity of the rib and ARROW layers. The first structure had the first oxide layer and an additional ARROW pair but did not have a rib etched into it. This device did not have measurable light propagation. We attribute this to the light being scattered off and absorbed by the walls of the trench.

The second test structure had a top membrane with a rib but no ARROW layers inside the trench. Again, no measurable light propagated beyond more than 1 mm. We again attribute this to the light being absorbed by the silicon trench. These two test devices show how critical both the rib and ARROW layers are for our devices to have effective low-loss propagation.

Finally, a destructive measuring technique called the cutback method was used to measure optical loss for the waveguides. We cleaved the waveguides to a given length, recorded optical transmission, then cleaved the waveguides at least twice more to shorter lengths and repeated the transmission measurement. Ideally, 1–2 mm of length was removed from the waveguide with each cleave. The transmission numbers were used to solve the loss coefficient, as shown by Equation (2).


(2) $${T\;=\;Ae}^{-\alpha x}$$

**Figure 6.**
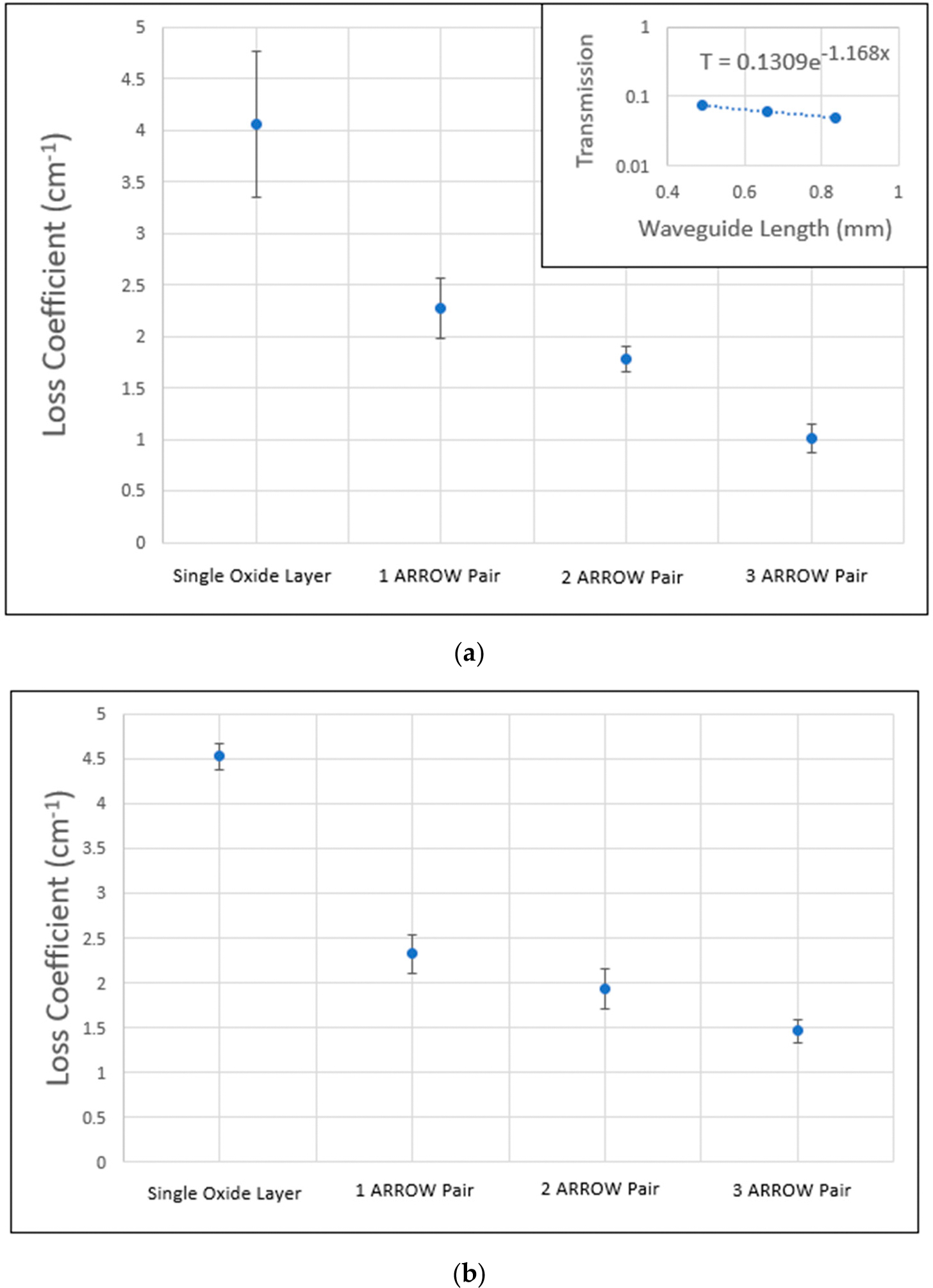
Measured optical loss for waveguide designs. (**a**) A 30 μm wide waveguide trench with the iteration order being the single oxide layer, and one, two and three ARROW layer pairs. A smaller plot shows the transmission vs. the waveguide length of a single chip. (**b**) A 20 μm wide waveguide trench with the same iteration order.

In this equation, T is the optical power transmitted through the waveguide while A is the optical power coupled into the waveguide. The loss coefficient is α and x the length of the waveguide. Transmission values were plotted versus x and fitted on a log plot to solve for α. Figure 6 shows the average loss for each design and how it decreases as we add more ARROW layer pairs. Figure 6a also shows an example of a transmission plot of a device. Typically, 12 to 24 waveguides were measured to obtain the reported loss coefficients. The error bars in the graph show the standard deviation of the data set.

Previous air-core ARROW designs that utilized a pedestal and surface micromachining exhibited an average loss coefficient of 1.54 cm^−1^ for a 15 μm wide device [18]. Our design measured an average loss coefficient of 1 cm^−1^ for a 30 μm wide waveguide and 1.4 cm^−1^ for a 20 μm wide waveguide. We expected the 30 μm wide device to guide with lower loss because the rib provides incomplete horizontal mode confinement and some of the light gets absorbed into the side walls. Since the side walls are further from the center and rib in the 30 μm wide design, we expected less interaction between light and sidewalls compared to the 20 μm wide design. Figure 6 shows how the loss coefficient decreases by adding ARROW pairs. A similar result was found with pedestal-based ARROWs [18].

Table 1 depicts multiple published designs of hollow ARROW waveguides that utilize various fabrication strategies. Some distinct differences in these designs are the use of wafer bonding, self-aligned pedestals, atomic layer deposition of the ARROW layers, and curved vs. square or rectangle waveguides. The size of the actual waveguide varies drastically with the larger sized cores typically producing lower loss.

## 5. Conclusions

We have shown that a hybrid structure combining a TIR-based rib with ARROW layers can create an effective hollow core waveguide in an etched trench structure. Four designs were detailed, and their overall loss compared. By adding a third set of ARROW layer pairs to the device, the loss coefficient was as low as 1 cm^−1^ for a mode on the order of 10 μm × 15 μm. The size of the mode was observed to change slightly in the horizontal and vertical direction as ARROW layer pairs were added, but the size of the mode was largely independent of the trench width for trenches etched 20 and 30 μm wide. This design is straightforward to fabricate and easily integrated into a variety of applications and photonic platforms.

## Figures and Tables

**Table 1.** Hollow ARROW waveguide design comparisons.

ARROW Waveguide Type	Optical Loss Coefficients	Cavity Size (μm)

Our Design	1 cm^−1^	15 × 20–30
Basic Hollow ARROW [17]	6.5 cm^−1^	3.5 × 12
SAP—Self Aligned Pedestal [18]	1.54 cm^−1^	5 × 15
SOC ARROW Single Over-Coating [21]	3.4 cm^−1^	5 × 12
Wafer Bonding [22]	0.465 cm^−1^ (2 dB/cm)	130 × 130
Atomic Layer Deposition [2]	5.22 cm^−1^	5 × 10
Curved Channels [23]	2.32 cm^−1^ (10 dB/cm)	5 × 100

## Data Availability

The related software code and further experimental data used in this paper are going to be open-sourced and available at BYU’s thesis and dissertation database. Please check the website at https://scholarsarchive.byu.edu/etd/.

## References

[1] Testa G; Persichetti G; Bernini R Liquid Core ARROW Waveguides: A Promising Photonic Structure for Integrated Optofluidic Microsensors. Micromachines 2016, 7, 47. 30407419 10.3390/mi7030047 PMC6190334

[2] Testa G; Huang Y; Zeni L; Sarro PM; Bernini R Liquid Core ARROW Waveguides by Atomic Layer Deposition. IEEE Photon-Technol. Lett. 2010, 22, 616–618.

[3] Unterkofler S; Garbos MK; Euser TG; Russell PSJ Long-distance laser propulsion and deformation-monitoring of cells in optofluidic photonic crystal fiber. J. Biophotonics 2012, 6, 743–752. 23281270 10.1002/jbio.201200180

[4] Napartovich AP; Elkin NN; Troshchieva VN; Vysotsky DV; Mawst LJ; Botez D Comprehensive Analysis of Mode Competition in High-Power CW-Operating Diode Lasers of the Antiresonant Reflecting Optical Waveguide (ARROW) Type. IEEE J. Sel. Top. Quantum Electron. 2011, 17, 1735–1744.

[5] Goddard NJ; Hulme J; Malins C; Singh K; Fielden PR Asymmetric anti-resonant reflecting optical waveguides (arrow) as chemical sensors. Analyst 2002, 127, 378–382. 11996363 10.1039/b109323a

[6] Russell PSJ; Hölzer P; Chang W; Abdolvand A; Travers JC Hollow-core photonic crystal fibres for gas-based nonlinear optics. Nat. Photon. 2014, 8, 278–286.

[7] Asha K; Krishnaswamy N; Suryanarayana NK Analysis of ARROW Waveguide Based Microcantilever for Sensing Application. Wirel. Pers. Commun. 2022, 126, 3435–3453.

[8] Ni W; Yang C; Luo Y; Xia R; Lu P; Hu DJJ; Danto S; Shum PP; Wei L Recent Advancement of Anti-Resonant Hollow-Core Fibers for Sensing Applications. Photonics 2021, 8, 128.

[9] Divakov DV; Lovetskiy KP; Мaлых МD; Tyutyunnik AA The application of Helmholtz decomposition method to investigation of multicore fibers and their application in Next-Generation communications systems. Lect. Notes Comput. Sci. 2018, 469–480.

[10] Chen J-H; Huang Y-T; Yang Y-L; Lu M-F; Shieh J-M Design, fabrication, and characterization of Si-based ARROW photonic crystal bend waveguides and power splitters. Appl. Opt. 2012, 51, 5876–5884. 22907016 10.1364/AO.51.005876

[11] Litchinitser NM; Dunn SC; Steinvurzel PE; Eggleton BJ; White TP; McPhedran RC; de Sterke CM Application of an ARROW model for designing tunable photonic devices. Opt. Express 2004, 12, 1540–1550. 19474979 10.1364/opex.12.001540

[12] Duguay A; Kokubun Y; Koch TL; Pfeiffer L Antiresonant reflecting opticalwaveguides in SiO_2_-Si multilayer structures. Appl. Phys. Lett. 1986, 49, 13–15.

[13] Walker ZJ; Wells T; Belliston E; Romney S; Walker SB; Sampad MJN; Saiduzzaman SM; Losakul R; Schmidt H; Hawkins AR Optofluidic Particle Manipulation Platform with Nanomembrane. Micromachines 2022, 13, 721. 35630187 10.3390/mi13050721 PMC9142978

[14] Asha K; Suryanarayana NK; Guha K; Iannacci J; Krishnaswamy N Modeling of photonic crystals anti resonant reflecting optical waveguide for sensing applications. Microsyst. Technol 2021, 27, 3859–3868.

[15] Wu B; Hulbert JF; Lunt EJ; Hurd K; Hawkins AR; Schmidt H Slow light on a chip via atomic quantum state control. Nat. Photon. 2010, 4, 776–779.

[16] Yin D; Schmidt H; Barber J; Hawkins A Integrated ARROW Wave Guides with Hollow Cores. Opt. Express 2004, 12, 2710–2715. 19475112 10.1364/opex.12.002710

[17] Integrated Microfabrication Lab (Cleanroom). Arrow Waveguide Layer Thickness Calculator. Available online: https://cleanroom.byu.edu/arrowcalc (accessed on 30 November 2023).

[18] Lunt EJ; Wu B; Keeley JM; Measor P; Schmidt H; Hawkins AR Hollow ARROW Waveguides on Self-Aligned Pedestals for Improved Geometry and Transmission. IEEE Photon-Technol. Lett. 2010, 22, 1147–1149. 10.1109/LPT.2010.2051145 PMC3059265 21423839

[19] Yin D; Barber JP; Hawkins AR; Schmidt H Waveguide loss optimization in hollow-core ARROW waveguides. Opt. Express 2005, 13, 9331–9336. 19503133 10.1364/opex.13.009331

[20] Walker Z; Wells T; Lay K; Sampad MJN; Schmidt H; Hawkins AR Solid-state membranes formed on natural menisci. Nanotechnology 2020, 31, 445303. 32679580 10.1088/1361-6528/aba711 PMC7931637

[21] Lunt EJ; Phillips BS; Keeley JM; Hawkins AR; Measor P; Wu B; Schmidt H Hollow ARROW waveguides on self-aligned pedestals for high-sensitivity optical sensing. In Proceedings of the SPIE 7591, Advanced Fabrication Technologies for Micro/Nano Optics and Photonics III, San Francisco, CA, USA, 16 February 2010; p. 759109.

[22] Bernini R; Campopiano S; Zeni L; Sarro PM ARROW optical waveguides based sensors. Sens. Actuators B Chem. 2004, 100, 143–146.

[23] Gollub AH; Carvalho D; De Paiva TC; Alayo MI Hollow core ARROW waveguides fabricated with SiOxNyfilms deposited at low temperatures. Phys. Status Solidi. C Conf. Crit. Rev 2010, 7, 964–967.

